# Vitamin D for Painful Diabetic Neuropathy: A Systematic Review and Meta‐Analysis of Randomised Controlled Trials

**DOI:** 10.1002/edm2.70118

**Published:** 2025-10-26

**Authors:** Abraham Gilbody, Joseph Gilbody

**Affiliations:** ^1^ North Bristol NHS Trust Bristol UK; ^2^ MRC Integrative Epidemiology Unit University of Bristol Bristol UK

## Abstract

**Background:**

Painful diabetic neuropathy (PDN) is a common complication of type 1 and type 2 diabetes, causing substantial morbidity. Current treatments, including antidepressants and analgesics, are often only partially effective and may have significant side effects. Vitamin D deficiency affects around 60% of people with diabetes, making supplementation a low‐toxicity, biologically plausible intervention. Observational studies suggest potential benefit, but robust evidence from randomised controlled trials (RCTs) is lacking.

**Objective:**

To systematically review and synthesise RCT evidence on the effect of vitamin D supplementation on pain outcomes in PDN.

**Methods:**

A systematic search was conducted in Medline, EMBASE, Web of Science, Cochrane Library, CINAHL, EBSCO and Google Scholar up to September 2025. Eligible studies were RCTs comparing vitamin D supplementation with placebo in adults with diabetes and PDN. The primary outcome was pain intensity measured with validated tools. A fixed‐effects meta‐analysis was performed, with results expressed as standardised mean differences (SMD). Risk of bias was assessed using the Cochrane RoB tool, and publication bias examined via Egger's funnel plot.

**Results:**

Five studies met inclusion criteria; four (*n* = 320) were included in the meta‐analysis. Pooled results showed a significant short‐term reduction in pain with vitamin D versus placebo (SMD = −0.85; 95% CI: −1.07 to −0.62), with moderate heterogeneity ( *I* ^2^ = 61.4%), equivalent to ~11 points on common pain scales. No studies reported medium‐ or long‐term outcomes. Study quality varied, with concerns regarding allocation concealment and selective outcome reporting. Four trials were prospectively registered.

**Conclusion:**

Vitamin D supplementation may reduce short‐term pain in PDN, but evidence is limited by small sample sizes and methodological quality. Larger, rigorously designed RCTs with longer follow‐up are needed before routine vitamin D testing or supplementation can be recommended for PDN in clinical practice.

## Graphical abstract

This systematic review and meta‐analysis of randomised controlled trials finds that vitamin D supplementation significantly reduces short‐term pain in patients with painful diabetic neuropathy. Findings support a potential low‐risk therapy, highlighting the need for larger trials to assess long‐term efficacy and guide future clinical guidelines.

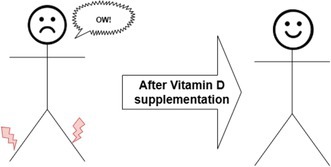

## 1 Introduction

Painful diabetic neuropathy (PDN) is a syndrome of sensory disorders caused by both type 1 and type 2 diabetes mellitus. PDN is known to be not only one of the most common diabetic peripheral neuropathies, but is also detrimental to the overall health of people with diabetes [1].

PDN is characterised by distressing tingling, burning, sharp, shooting and lancinating or even electric shock sensations [2], with nocturnal exacerbation and impacts on sleep. PDN also contributes to diabetic foot disease by causing poorly healing ulcers and eventual amputation [3]. This contributes to reduced physical activity, depression and decline in quality of life (QOL) [4].

Epidemiological data demonstrates that the incidence of pain symptoms in diabetes is 10%–20% and 40%–50% with diabetic neuropathy [5, 6, 7]. PDN also contributes to higher rates of healthcare utilisation and costs [8]. Patients with PDN have increased costs due to more frequent and prolonged hospitalizations and increased rates of outpatient visits, coupled with reduced economic productivity and sickness absence [8].

Treatment of PDN comprises three approaches: (1) intensive glycaemic control and risk factor management, (2) treatments based on pathogenetic mechanisms and (3) symptomatic pain management [9]. Intense glucose control is currently the only intervention that has been proven to reduce the risk of development of neuropathy [10, 11]. Peripheral nerve injury is largely understood as the main source of pain; however, recent literature supports the role of the central nervous system in the disinhibition and amplification of pain [12].

Consequently, clinical guidelines recommend symptomatic relief of neuropathic pain through the ‘centrally acting’ analgesic effects of antidepressant drugs (tricyclic antidepressants or selective serotonin reuptake inhibitors), anticonvulsants and opioids [13]. Treatment of PDN is far from satisfactory, with many medications providing limited pain relief and a significant side effect profile [9].

Another treatment modality emerging is the use of vitamin D, which will be the focus of this systematic review and meta‐analysis. Vitamin D deficiency has been shown to be an independent risk factor for the development of PDN [14]. Vitamin D has also been shown in some studies to have a significant influence in PDN compared to painless DPN [15]. Additionally, individuals with PDN are more likely to have vitamin D deficiency versus those with painless diabetic neuropathy [16]. Furthermore, non‐randomised evidence shows that high‐dose intramuscular injections of vitamin D can reduce pain [17]. Vitamin D is thought to act on PDN through neurotrophic, anti‐inflammatory and nociceptive pathways [18, 19, 20]. This supports the biologically plausible idea that correcting vitamin D deficiency could have therapeutic effects in PDN. However, definitive evidence will only emerge from robust controlled clinical studies. Research intelligence is needed to establish whether vitamin D therapy treats or delays the onset of symptoms in painful diabetic neuropathy. At the time of writing, there was no large‐scale RCT or long‐term follow‐up studies on the effect of vitamin D therapy on the treatment of PDN, highlighting the necessity of further data synthesis. Another treatment modality is emerging is the use of vitamin D which will be the focus of this systematic review, and meta‐analysis. Vitamin D deficiency been shown to be an independent risk factor for the development of PDN [14]. Vitamin D has also been shown in some studies to have a significant influence in PDN compared to painless DPN [15]. Additionally, individuals with PDN are more likely to have vitamin D deficiency versus those with painless diabetic neuropathy [16]. Furthermore, non‐randomised evidence shows that high‐dose intramuscular injections of vitamin D can reduce pain [17]. The vitamin D is thought to act on PDN through neurotrophic, anti‐inflammatory and nociceptive pathways [21]. This supports the biologically plausible idea that correcting vitamin D deficiency could have therapeutic effects in PDN. However definitive evidence will only emerge from robust controlled clinical studies. Research intelligence is needed to establish whether vitamin D therapy treats or delays the onset of symptoms in painful diabetic neuropathy. At the time of writing there was no large scale RCT or long term follow‐up studies on the effect of vitamin D therapy on the treatment of PDN highlighting the necessity of further data synthesis to guide future research.

## 2 Review Aim

The overarching aim of this systematic review was to synthesise data from randomised controlled trials of vitamin D supplementation and its effects on painful diabetic neuropathy.

By using systematic review methods, a supplementary aim was to map the strengths and limitations of existing available research, and to make recommendations for clinical practice and identify the need for further research.

## 3 Review Methods

The systematic review was undertaken in September 2025 in accordance with the Methods of the Cochrane Handbook for Systematic Reviews [22], and followed the Preferred Reporting Items for Systematic Reviews and Meta‐Analyses (PRISMA) guidelines [23].

### 3.1 Search Strategy

A search strategy was devised to identify relevant studies from the following electronic databases: Medline, EMBASE, Web of Science, Cochrane Library, CINAHL, EBSCO and Google Scholar. These databases were selected, as recommended by the Cochrane Handbook, as MEDLINE, Cochrane Central and Embase are effective in identifying randomised controlled trials [22]. The additional databases facilitate a wider and more extensive search of sources, thus minimising selection bias for the studies [22]. Search terms were devised from the PICO structure [described below] and utilised optimal Cochrane strategies to identify randomised controlled studies [24], whilst excluding non‐randomised data (a sensitive and specific search). Resources were only available for English language publication; therefore, the search was restricted. See Appendix 2 for search MeSH terms and strategies.

In addition to the electronic bibliographic searches, reference lists from included papers were scrutinised to identify additional studies [‘backwards citation searching’]. Additional studies citing included studies were also sought using Google Scholar [‘forward citation searching’] [25].

### 3.2 Criteria for Study Selection

Only randomised controlled trials (RCTs) were considered as they are considered the gold standard design for studying cause–effect relationships between an intervention and outcome. Randomization is the optimum design in eliminating much of the bias inherent with other study designs, and [most importantly] minimises the distortion of cause–effect from both known and unknown confounding factors [26]. Non‐randomised controlled studies, reviews and case‐series were excluded. Potentially eligible studies were assessed for inclusion against the PICO criteria for inclusion in the systematic review and are summarised in Table 1.

**TABLE 1:** study inclusion criteria for systematic review.

PICO criterion	How defined in review
Participants	Patients with diabetes (type 1, type 2) were selected for inclusion; both with and without vitamin D deficiency. Diabetes had to be diagnosed against an accepted international criterion. No restrictions were placed according to patient age or location. Studies were included if there was pre‐existing painful diabetic neuropathy. The operational definition of PDN followed the definition of neuropathic pain by IASP (International Association for the Study of Pain). According to these guidelines, PDN is pain arising as a direct consequence of abnormalities in the somatosensory system in people with diabetes
Intervention	Studies were included where vitamin D was administered via any route. Note was taken of the route [oral, or parenteral], total dosage of vitamin D and this was compared with reference to BNF guidelines. Studies were included where vitamin D was administered alone or in combination with another drug
Comparator	Studies had to include a comparator group to facilitate meaningful comparison to be made between treated and untreated groups. We prioritised studies that compared vitamin D with an inert placebo
Outcomes [measure and duration]	Pain was selected as the primary outcome of interest. To be included a study had to use a validated pain assessment tool. For example generic pain questionnaires such as the McGill Pain Questionnaire [27] and Diabetic Neuropathic specific questionnaires such as *Douleur Neuropathique en 4 Questions* (DN4) questionnaire [28] for the assessment of PDN pain outcomes Outcomes were short term (≤ 3 months), medium term (> 3 months to ≤ 6 months) and longer term (> 6 months)

### 3.3 Data Collection and Extraction

Relevant data were first extracted into a spreadsheet. Relevant data were: the mean pain scores, % change in pain score, dosage and type of vitamin D supplementation used, standard deviations, sample sizes and patient gender in both intervention and control groups. Study authors were contacted to provide missing data; however, no additional data were obtained.

### 3.4 Quality Assessment of Trials

As per the Cochrane Handbook (21), trials were assessed on quality based on 3 main parameters (greater detail in Appendix 1):

- Randomization (allocation sequence generation and allocation sequence concealment present).
- Blinding (double blinding, effective blinding, bias due to measurement of outcomes, bias due to missing outcome data).
- Attrition bias (loss to follow up, correct analysis preformed).

Each parameter underwent a risk‐of‐bias judgement and was assigned to 3 groups:

- Low risk‐of‐bias.
- Moderate risk‐of‐bias.
- High risk‐of‐bias.

The overall judgement of bias was based on the collection of ratings. Quality assessed data were plotted into the risk of bias (RoB) tool found on Revman5 (see Table 2). This presented the overall judgement of risk of bias using an infographic. The GRADE tool was used to assess the quality of the data from each study using several domains. Study quality was marked down for: risk of bias, imprecision, inconsistency, indirectness and publication bias. Marked up for: magnitude of effect, dose‐dependent gradient and all residual confounding would decrease magnitude of effect.

**TABLE 2:** Overall risk‐of‐bias judgment criteria.

Overall risk of bias judgement	Criteria
Low risk of bias	The study is judged to be at low risk of bias for all domains for this result
Moderate risk of bias	The study is judged to raise moderate risk of bias in at least one domain for this result, but not to be at high risk of bias for any domain
High risk of bias	The study is judged to be at high risk of bias in at least one domain for this result or The study is judged to have moderate risk of bias for multiple domains in a way that substantially lowers confidence in the result

The criteria for Cochrane for randomization [Cochrane Handbook for Systematic Reviews of Interventions 4.2.5 (21)] are reproduced in Appendix 1.

### 3.5 Small Study, Publication and Outcome Reporting Bias

A range of databases was searched to minimise study identification and selection biases. Publication bias arises through the inclusion of only published studies and the potential omission of negative studies since these are often unpublished [22]. A funnel plot analysis was planned to detect if authors publish smaller studies with positive results [22]. This is commonly found among studies that are smaller in size and are typically performed with reduced methodological rigour, producing an overestimation of the intervention effect [22]. An asymmetrical funnel plot indicates that smaller studies were subject to publication and small study bias. The greater the asymmetry, the increased likelihood that the amount of bias will be substantial. To quantify the magnitude of funnel plot asymmetry, an Egger test was planned [29].

Selective reporting of outcome bias was addressed (where possible) by comparing the outcomes described in the study trial register or pre‐study protocol with the range of outcomes reported in the final study publication [30].

### 3.6 Proposed Meta‐Analysis

In line with Cochrane guidance, where similar [non‐heterogeneous] data were available for three or more studies, then a fixed effects meta‐analysis was undertaken using RevMan 5 software [31]. It was anticipated that the primary pain outcome would be presented as a continuous variable of change in pain scores in both control and intervention groups. Endpoint data in each group were combined to calculate between‐group differences. Where trials used different instruments to measure the pain outcomes, a standardised mean difference (SMD) with 95% confidence intervals was calculated. The standardised mean difference measures the size of the intervention effect of each study compared to the between‐participant variability in outcome measurements recorded in each individual study. To allow for easier interpretation and analysis of the results, we planned to convert the SMD into the specific clinical measures used in the studies (such as the McGill Pain Questionnaire or the Leeds Assessment of Neuropathic Symptoms and Signs [LANSS] scale). This can be done by multiplying the SMD by an estimate of the standard deviation (SD) associated with the outcome measure instrument, according to the Cochrane Handbook.

### 3.7 Assessment of Heterogeneity

Heterogeneity was tested using Higgins' test (*I* ^2^) to test for between‐study variation in order to determine if studies can be pooled for meta‐analysis [32].

- If < 75% use fixed effects.
- If more than 75% use random effects.
- If more than 90% just use systematic narrative review (no meta‐analysis undertaken).

Where significant heterogeneity was found, sources of heterogeneity were explored. Anticipated source of heterogeneity included: dose and route of vitamin D; baseline vitamin D status of participants, duration of follow up.

## 4 Results

### 4.1 Study Selection

**FIGURE 1:**
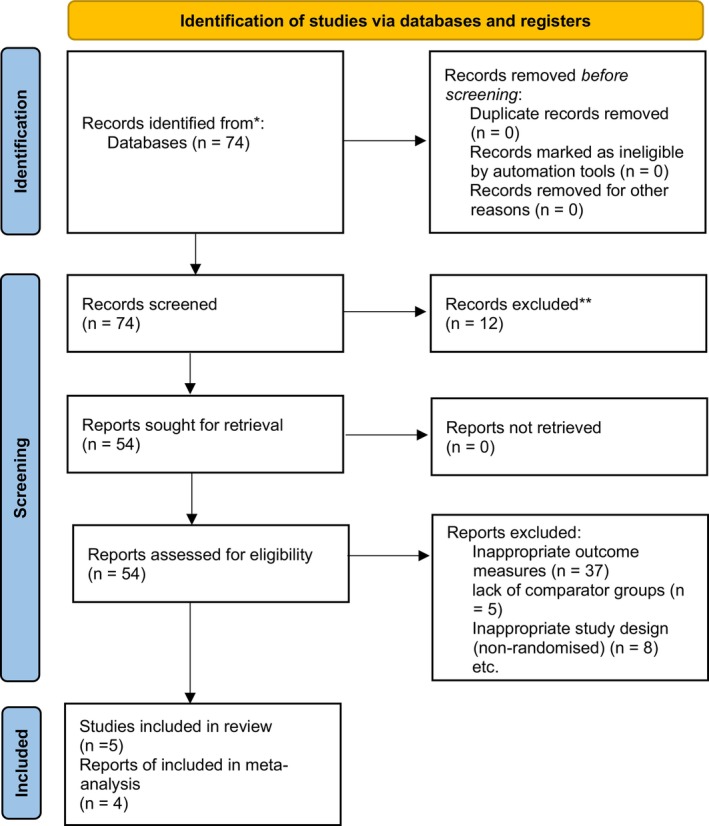
PRISMA flow diagram.

The selection process is summarised using the PRISMA flow diagram (Figure 1). The literature search identified a total of 73 potentially eligible studies. After the removal of duplicates, 63 papers were included in the initial screening procedure. Electronic titles and abstracts of papers from the search were first screened for eligibility. Following the removal of 12 papers, the remaining papers were screened on their full text against PICO criteria detailed below. Where the full text was not available, the study's corresponding author was contacted via email. In 5 papers, it was found that they were eligible for use in the systematic review [33, 34, 35, 36, 37] but only four of those provided sufficient data for the meta‐analysis [33, 34, 35, 37], with the 5th excluded due to the lack of reporting of pain outcomes for just vitamin D supplementation. Common reasons for exclusion included inappropriate outcome measurements, lack of comparator groups and inappropriate study design (non‐randomised).

Four studies that were randomised and met the PICO inclusion criteria [33, 34, 35, 37] were eligible for inclusion in the meta‐analysis. One study was a five‐arm trial, and two arms provided data that allowed a comparison between vitamin D and placebo. Study size ranged from 60 to 112 and 320 participants were eligible for meta‐analytic pooling. The weighting of each paper in the meta‐analysis was proportional to their respective participant totals (and inversely related to the variance). Each study reported different pain scales, with the visual analogue scale [35], the NSS [33] and the Neuropathic pain scale (NPS) [34]. Each study recruited patients who were vitamin D deficient at baseline, with placebo arm levels ranging from 11.68 ng/mL (SD 3.8) to 24.6 ng/mL (SD 5.5) [34]. Vitamin D was administered orally in all cases, and dose regimens ranged from 4000 IU per day to 50,000 IU every week. Duration of follow‐up ranged from 8 weeks [33, 35, 37] to 12 weeks [34]. For PICO and study details, see Table 3.

**TABLE 3:** Data extracted for studies meeting PICO including criteria.

Study	Design	Participant characteristics	Pain outcome	Intervention	Comparator
Shehab et al. (2015)	Placebo‐controlled randomised trial	*N* = 112 participants (placebo = 55, vitamin D = 57) Sex: Placebo (M 27 F 28) Vitamin D (M 21 F 36) Age: Placebo (59.8 years SD = 9.6) Vitamin D (61.8 SD = 8.1) Baseline vitamin D: Placebo (29.2 nmol/mL SD 9.5) (11.68 ng/mL SD 3.8) Vitamin D (25.3 nmol/mL SD 10.9) (10.12 ng/mL SD 4.36) Final vitamin D level: Placebo (30.3 nmol/mL SD 8.9) (12.12 ng/mL SD 3.56) Vitamin D (58.2 nmol/mL SD 23.8) (23.28 ng/mL SD 9.52) Change in vitamin D level: Placebo (1.1 nmol/mL SD 3.6) (0.44 ng/mL SD 1.44) Vitamin D (32.8 nmol/mL SD 23.7) (13.12 ng/mL SD 9.48) Period of follow up: 8 weeks Diagnosis: Adult patients with type 2 diabetes with DPN and vitamin D deficiency	Primary outcome Neuropathic Symptom Score (NSS) Placebo: pre‐test (5.50 SD = 1.25) after test (5.45 SD = 1.20) difference (−0.20 SD 0.59) Treatment: pre‐test (5.92 SD 1.29) post‐test (4.43 SD 1.58) difference (−1.49 SD 1.37) *p* < 0.001 Outcome measured at 8 weeks	The treatment group received oral capsules of vitamin D3 (a capsule of cholecalciferol, 50,000 IU, Bronson, Lindon, Utah, USA) once weekly for 8 weeks	The placebo group received starch capsules, also once weekly for 8 weeks
Pinzon et al. (2021)	Randomised controlled clinical trial	*N* = 68 (placebo = 34 vitamin D = 34) Sex: Female 41 (60.3%) Male 27 (39.7%) Age: mean: 64.96 ± 8.3 Baseline vitamin D level: Placebo (15.62 ng/mL SD 8.69) Vitamin D (15.87 ng/mL SD 8.50) Week 8: Placebo (18.73 ng/mL SD 6.88) Vitamin D (40.02 ng/mL SD 15.33) Change in vitamin D level: Placebo (3.10 ng/mL SD 4.20) Vitamin D (24.14 ng/mL SD 13.68) Period of follow up: 8 weeks Diagnosis: Adult patients with type 2 diabetes with DPN and vitamin D deficiency	Visual analogue scale Placebo: pre‐treatment (5.46 SD 2.13) post‐treatment (3.09 SD 2.33) difference (−2.37 SD 2.2) Vitamin D: pre‐treatment (5.74 SD 2.16) post‐treatment (2.39 SD 2.09) difference (−3.34 SD 2.03)	Experimental group received an oral vitamin D 5000 IU (Hi‐D 5000) once daily over 8 weeks in addition to the standard treatment (pregabalin 1 × 75 mg, gabapentin 1 × 100 mg, or amitriptyline 1 × 25 mg; the dose was adjusted according to each patients' symptom) for diabetic neuropathy	The control group received standard treatment only over the same period
Davoudi et al. (2021)	RCT (five arms), including vitamin D and placebo arms (used in this analysis)	Total 225 participants. 90 participants selected (45 placebo (5 dropouts) 45 vitamin D (5 dropouts) Sex: Placebo (40% F) vitamin D (55% F) Age: Placebo (56.24 ± 9.89) Vitamin D (54.5 ± 9.8) Baseline vitamin D level: Placebo (24.6 ng/mL SD 5.5) Vitamin D (27.4 ng/mL SD 5.07) Change in vitamin D level: Not reported Period of follow‐up: 12 weeks Diagnosis: Adult patients with type 2 diabetes with neuropathy and vitamin D deficiency	Neuropathic pain severity: Placebo: Pre‐treatment (47.02 SD 13.7) Post‐treatment (45.6 SD 13.4) Vitamin D: Pre‐treatment (49.4 SD 14.7) Post‐treatment (31.03 SD 14.8)	Daily 4000 IU oral dosage (four capsules) with 28,000 IU vitamin D weekly for 12 weeks	Placebo plus usual treatment based on neurologist prescribes
Pinzon et al. (2025)	Randomised controlled, open‐label trial with two groups	A total of 60 subjects were included, with 30 in the experimental group and 30 in the control group Sex: 60% men and 40% women in both groups Age: Experimental group (62.56 ± 6.272), control group (65.04 ± 9.449) Diagnosis: Adult patients with painful diabetic neuropathy and type 2 diabetes	Pain severity (Visual Analog Scale 0–10): Placebo: Pre‐treatment (5.38 ± 2.13) Post‐treatment (3.11 ± 2.33). Vitamin D: Pre‐treatment (5.62 ± 2.16) Post‐treatment (2.25 ± 2.09)	Experimental group received an oral dose of 2000 IU/day, given as 1000 IU twice daily, for 8 weeks	Both groups received standard symptomatic therapy (gabapentin, pregabalin or amitriptyline)

Abbreviations: f, female participants; IU, internation unit (the amount of a substance that has a certain biological effect); m, male participants; *n*, number of participants; SD, standard difference.

**FIGURE 2:**
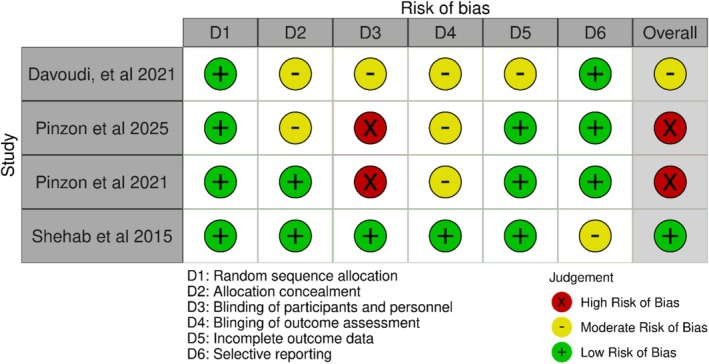
Cochrane risk of bias assessment.

With respect to study quality, all studies were rated using the GRADE criteria to assess for overall risk of bias. One study [33] maintained an overall low risk of bias by adhering to stringent experimental techniques to minimise bias. Three studies [34, 35, 37] were moderate or high risk of bias due to omitting key techniques such as concealment of allocation and effective double blinding. It is not clear what the impact of this limitation may have on the observed effect sizes; however, sub‐par allocation concealment is generally associated with the exaggerated inflation of an effect relative to the true effect [38]. For a full summary of quality assessment criteria, see Table 4 and a summary of Cochrane risk of bias, see Figure 2 [below].

**TABLE 4:** Quality assessment of studies using Cochrane risk of bias tool.

Study	Randomization	Blinding	Attrition
	Sequence generation	Masking of study participants and personnel	Outcome data not available
	Allocation concealment (random sequence)		
Shehab et al. (2015) Overall: Low risk‐of‐bias	Did not explicitly state randomised, but participants allocated to groups according to a sequential order generated by the hospital recording system Concealed opaque envelopes were prepared for all study participants for allocation into the vitamin D treatment group or the placebo group. This was done under the direct control of a single investigator (H.M.) Low risk‐of‐bias	Double‐blinded study The study participants and the outcome assessor were blinded to the treatment allocation until the conclusion of the study Low risk‐of‐bias	Two patients dropped out of the treatment group and 3 out of the placebo group These dropouts are acknowledged however are not accounted for in the analysis. 95% of participants analysed so considered low risk No intention to treat Low risk of bias
Pinzon et al. (2021) Overall: Moderate risk‐of‐bias	Randomization was carried out using block randomization with a 1:1 ratio (allocation of participants may be predictable and result in selection bias when the study groups are unmasked) A randomization list was generated by a statistician not involved with the study, using blocks of 5 stratifications No concealment of allocation mentioned however allocation performed by an external assessor Low risk‐of‐bias	Complete blinding was considered difficult and not possible. Participants were informed of key elements of the respective intervention and follow‐up they were randomised to, but not on information about the treatment and follow‐up alternatives in the other group or the study's hypotheses Complete blinding not performed. Participants were only informed of the intervention for the group they were allocated to Moderate risk‐of‐bias	All participants who were lost to follow‐up were accounted for in the consort flow diagram Intention to treat analysis used Low risk‐of‐bias
Davoudi et al. (2021) Overall: moderate risk‐of‐bias	Participants were randomly allocated into groups using a random table. No further details given No concealment of allocation mentioned Moderate risk‐of‐bias	All patients were blinded to receive VD or placebo groups Single blind No mention of blinding of assessment of outcome Moderate risk of bias	Toward covering potential dropouts, the sample size increased 25% and reaches to 45 subjects for each group Less than 95% participants analysed and no ITT analysis performed Patient dropouts are not acknowledged in consort flow diagram and not accounted for in the statistical analysis Moderate risk‐of‐bias
Pinzon and Angela (2025)	The study was a randomised controlled trial that used a computerised block randomization technique. The method of allocation concealment was not explicitly described	The study was open‐label, and measurements were carried out by trained enumerators who were not blinded. The authors noted this as a limitation, as it may ‘exaggerate the estimates of treatment effects’	The study calculation assumed a 10% loss of follow‐up, and the analysis was conducted using the principle of intention to treat. No dropouts were reported in the results
Overall: Moderate risk‐of‐bias	YModerate risk‐of‐bias	High risk‐of‐bias	Low risk of bias from attrition

### 4.2 Meta‐Analysis

**FIGURE 3:**
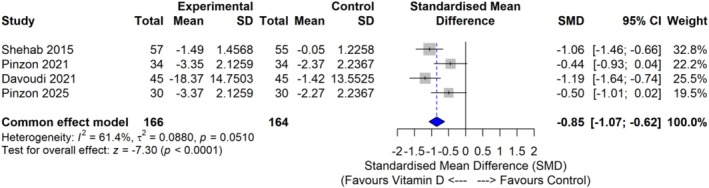
Forest plot of a fixed effect meta‐analysis of the effect of vitamin D on short‐term outcomes.

**FIGURE 4:**
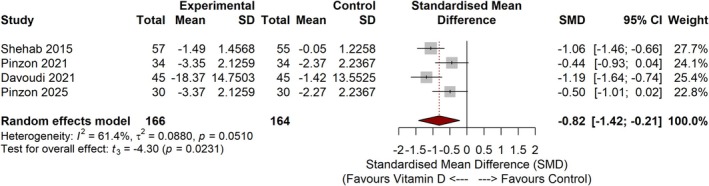
Forest plot of a random effects meta‐analysis of the effect of vitamin D on short‐term outcomes.

A fixed effects meta‐analysis was performed using RevMan 5. All studies were positive [favouring vitamin D]; each included study used a different pain assessment tool: the Visual Analogue Scale (VAS; range: 0–10), Neuropathic Pain Scale (NPS; range: 0–100) and Neuropathy Symptom Score (NSS; range: 0–9). These were standardised for meta‐analysis using the pooled standard deviation to allow comparability across scales. Standard effect sizes ranged from SMD = −1.19 (95% CI: −1.50 to −0.57) [34] to SMD = −0.44 (95% CI: −0.79 to 0.16) [35]. The overall pooled effect size was strongly in favour of vitamin D supplementation (pooled fixed effect standard effect size = −0.85; 95% CI: −1.07 to −0.62). There was moderate to substantial between study heterogeneity (Higgins *I* ^2^ = 61.4%), meaning a random effects model was also used, which also showed a strong reduction in pain outcomes (−0.82; 95% CI: −1.42 to −0.21). See Figures 3 and 4.

The standard effect size of −0.70 was moderate in size and equivalent to a reduction of 11.6 (95% CI: 15.1–8.2) points on the Neuropathic Pain Scale [assuming SD of 13.7, derived from the placebo arm baseline data].

### 4.3 Test for Small Study Bias

**FIGURE 5:**
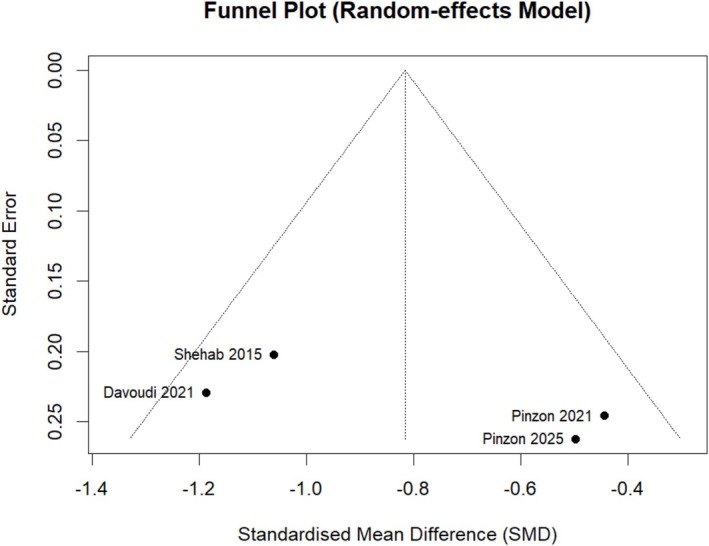
Funnel plot to test for small study bias among included studies.

There were four eligible studies, with little variation in sample size, making the Egger forest plot difficult to interpret. The effect of publication and small study bias was therefore difficult to exclude. See Figure 5 for the Funnel plot. The Egger test was not performed due to limited sample size.

### 4.4 Long Term Outcomes

None of the studies included follow‐ups beyond 12 weeks, so no further analyses on this are included in this review.

### 4.5 Selective Reporting Bias

Two studies were pre‐registered and the reported pain outcomes in the final manuscript corresponded with the study protocol [34, 35]. One study was unregistered and there was no available public domain protocol [33].

## 5 Discussion

### 5.1 Summary of Main Results

Randomised trials were available to answer the main review question. Four small‐scale trials showed a reduction in the painful symptoms of peripheral diabetic neuropathy, regardless of the pain outcome used. When the data were pooled, the meta‐analysis results demonstrated a significant statistical improvement in reported neuropathic pain by participants. This strongly favours vitamin D supplementation for the reduction in painful symptoms of diabetic neuropathy compared to control treatment in all trials.

Interpreting the pooled standardised mean difference (SMD = −0.85) in clinical terms, this represents a moderate‐to‐large effect. Although the included studies used different pain scales (VAS, NPS and NSS), each reported reductions in pain scores that were directionally consistent and of a magnitude likely to reflect clinically meaningful improvement. Importantly, the observed changes either exceeded or closely approached commonly accepted thresholds for the minimal clinically important difference (MCID) on each respective scale. This supports the conclusion that vitamin D supplementation may provide a tangible benefit for patients with painful diabetic neuropathy, despite variation in outcome measurement. Both the random and fixed effects models identified a very similar effect estimate, indicating that heterogeneity between the studies was not a big factor in the estimate.

### 5.2 Agreements and Disagreements With Other Studies or Reviews

The review findings are consistent with previous non‐experimental studies that have reported vitamin D as an independent risk factor in peripheral neuropathy in patients with low vitamin D [14]. Systematic reviews of non‐experimental studies have found consistent evidence for associations of vitamin D deficiencies with PDN [18]. Non‐randomised studies have established that correction of vitamin D deficiency leads to an improvement in symptoms [17, 39], and this is the first time this has been demonstrated in the context of a meta‐analysis of RCT data. A meta‐analysis of a mixture of randomised and non‐randomised interventions showed similar results, with vitamin D ameliorating patients' pain [40]. It is important to note, however, that benefits may not extend beyond correction of deficiency; trials in replete populations are needed. It is also difficult to establish if the effects of vitamin D supplementation were due to an elevation in the pain threshold, due to improvement of affected nerves, or via improvement in diabetic markers (HbA1c) or a synergy of them all [39, 41, 42].

There are plausible biological mechanisms by which this reduction in pain can be explained. Vitamin D is a potent inducer of neurotrophins and neurotransmitters; nerve growth factor (NGF) is such an example [41]. Vitamin D supplementation has been shown to increase nerve growth factor (NGF), a protein required for nerve growth and maintenance in the peripheral nervous system [41]. Epidermal keratinocytes are the primary source of NGF in the skin; lower dermal levels of NGF in patients with diabetic polyneuropathy are associated with neuropathic signs of sensor and autonomic nerve function. This suggests that the diminished levels of NGF in epidermal keratinocytes contribute to the pathogenesis of peripheral neuropathy [41].

Furthermore, there is a suggested mechanistic association between neuropathic pain and vitamin D through nociceptive calcitonin gene‐associated peptide (CGRP)‐positive neurons that have been shown to have a distinct vitamin D phenotype alongside hormonally controlled receptor and ligand levels [35, 43].

### 5.3 Applicability of Evidence

Correcting vitamin D levels in patients where there is deficiency is already clinically indicated. However, the observed effects in this preliminary meta‐analysis demonstrate the potential added benefit of reduction in painful symptoms of PDN. On top of this, correction of vitamin D levels is a comparatively low‐risk intervention that has also been related to lowering HbA1c levels, reducing insulin resistance and improving insulin sensitivity [42, 44]. Therefore, there is the potential for vitamin D to not only relieve painful symptoms but also improve glycaemic regulation.

Reduction in pain severity and pain disability has many benefits including an improvement in QOL of patients through better outcomes in sleep quality, general activity and mood symptoms [45]. Individuals with reduced pain severity are less likely to incur healthcare costs through the use of polypharmacy and increased outpatient visits along with increased economic productivity [8, 45]. The widespread use of vitamin D as an adjuvant medication in the control of the symptoms of PDN could therefore be useful in mitigating the high economic and healthcare burden associated with diabetic complications [46].

Of note is that all four of the studies recruited participants who reported a baseline vitamin D deficiency. This remains highly applicable to many patients with painful diabetic neuropathy since vitamin D deficiency is commonly observed across diabetic populations, with one study finding 89% of participants were found to be deficient in vitamin D, with just 9 in 300 participants having sufficient levels of vitamin D [47]. One limitation to the findings is the specificity of the results to those patients with an underlying vitamin D deficiency, with the outcome effects being as a result of correcting this pre‐existing deficiency. This provides limitations to the applicability of these results to non‐vitamin D deficient patients with PDN, as further study of non‐deficient patients would be required.

It is difficult to make firm conclusions for the applicability of this evidence. However, one important clinical implication is the need for patients with PDN to be routinely tested for vitamin D levels and to have a low threshold for prescribing vitamin D for patients at risk of deficiency. Furthermore, the indicated pharmaceutical benefits of vitamin D are significant due to its many perceived advantages over traditional pharmaceutical treatment for diabetic neuropathy. Vitamin D is a relatively low cost, meaning it could be a useful adjunct to existing treatments with the potential to demonstrate likely cost effectiveness by offsetting the need for prescriptions of expensive analgesic drugs or fewer iatrogenic side effects of high dose analgesic medicines.

According to the NICE treatment pathways for neuropathic pain in adults, vitamin D is not currently recommended due to its limited evidence base [48]. In light of this review, vitamin D therapy demonstrated a significant effect that might be useful in the formulation of future guidelines.

### 5.4 Quality of the Evidence

The risk of bias assessment indicated a variation in the overall assessment of each study included. There was moderate to high risk of bias for 3 of the studies for the category describing bias in the blinding process. This was typically because of suboptimal blinding process or only single blinding occurring. In the domain of bias due to missing outcome data, most studies were found to have a low risk of bias. However, one study was found to have a moderate risk of bias; this was due to failure to perform intention‐to‐treat analysis. The impact of the missing data on the observed effects of this study was unclear. In the final domain, bias in the selection of the reported outcome, most papers were reported as having a low risk of bias; however, in one, there was an absence of a pre‐registered report of trial details, thus making it a moderate risk of bias. This makes it difficult to make sure that the reported analysis was consistent with the pre‐planned analysis.

None of the studies reported outcomes beyond 12 weeks, highlighting a major gap in the literature. To the best of our knowledge, no ongoing trials are currently addressing this issue. Future research should incorporate longer follow‐up periods to evaluate long‐term outcomes. One limitation of the studies included was the large variation in the prescribed vitamin supplementation, ranging from a single weekly oral dose of 50,000 IU vitamin D to a daily dose of 5000 IU. Furthermore, analysis was hampered by an overall small sample size (*n* = 260) over the 3 papers, limiting both statistical power and generalizability. Additionally, the heterogeneity in pain measurement scales across the four studies may introduce variability that complicates the interpretation of pooled effect estimates in the meta‐analysis. These collectively could lead to an inflation of effect estimates. There is an urgent need to replicate the findings of this review, based on small‐scale studies, in a large‐scale randomised controlled trial with sufficient statistical power, using standardised protocols for both vitamin D dosing and pain scoring. Tests for small study bias using funnel plots were difficult to interpret, and there is a risk that these results might be overturned by a large‐scale trial that is free from publication bias.

### 5.5 Implications for Research

These preliminary results on a relatively small sample size confirm the need for a large fully powered study with a concurrent cost effectiveness analysis to explore whether vitamin D therapy represents good value for money. Mechanisms by which it might demonstrate cost effectiveness include decreased need for polypharmacy, particularly expensive and potentially toxic painkiller medications. This is because the cost of treating diabetic neuropathy is very high and is therefore very important to find the most effective and cost‐effective medications [8].

Future research should centre around whether these results are applicable to patients suffering from PDN who do not have underlying vitamin D deficiency and ascertain whether clinical findings are due to the correction of underlying vitamin D deficiency. Furthermore, future research on the proper dosing in addition to the route and duration of treatment is essential to further explore the efficacy and clinical benefits of the use of vitamin D supplementation for PDN and effectively help advise policy on the dispensation of the drug.

Secondary objectives assessing the effect of vitamin D supplementation on glycaemic control should also be addressed.

If vitamin D supplementation proves to be an effective and scalable treatment for PDN, it could substantially reduce the global disease burden associated with this condition.

## Author Contributions

The meta‐analysis was conducted by Abraham Gilbody. All authors contributed to the quality assessment and selection of studies for inclusion. Statistical and epidemiological expertise was provided by Dr Joe Gilbody.

## Conflicts of Interest

The authors declare no conflicts of interest.

## Data Availability

The data that support the findings of this study are openly available in PubMed at https://pmc.ncbi.nlm.nih.gov, reference number 8625262, 25720672, 34984028.

## Appendix 1 Criteria for the Quality Assessment of Trials Using the Cochrane Risk of Bias Tool

**Table:**

Randomization & concealment of allocation	Blinding	Attrition
This will be adequate when patients are allocation into study groups via a method that used numerical randomization (allocation sequence randomization). Furthermore, the allocation of study participants must be concealed to both them and trial team members until it is completed. This is to avoid selective enrolment of participants based on the treatment assignment (allocation sequence concealment)	Adequate when double‐blinding has occurred, this includes masking of the treatment allocation to both the participants and the trial members. This ensures there is a decreased risk of both deviance from the application of the treatment and the use of non‐protocol medication alongside the trial medication (double blinding)	Adequate when missing outcome data are taken into account in the final analysis and whether the missingness mechanism is linked to the value of the true outcome. Furthermore, the proportion of dropouts must be minimal for the results to be weighted in our analysis. This will avoid bias due to mechanics of the study altering the dropout rate dependant on the intervention given

## Appendix 2 Search Strategies to Identify RCTs of Vitamin D for Preventing Peripheral Diabetic Neuropathy

### MEDLINE (via PubMed)

We combined Cochrane RCT‐specific search filters with the following terms.

((diabetes mellitus[MeSH Terms] OR diabet*[tiab])

AND (peripheral neuropathy[MeSH Terms] OR neuropath*[tiab])

AND (“vitamin D”[MeSH Terms] OR “vitamin D”[tiab] OR cholecalciferol[tiab] OR ergocalciferol[tiab] OR calcifediol[tiab] OR “25(OH) D”[tiab] OR “25‐Hydroxyvitamin D”[tiab]))

### EMBASE (via Ovid)

We combined Cochrane RCT‐specific search filters with the following terms.

((diabetes mellitus/OR diabet*.ti, ab.)

AND (peripheral neuropathy/OR neuropath*.ti,ab.)

AND (vitamin D/OR “vitamin D”.ti,ab. OR cholecalciferol.ti,ab. OR ergocalciferol.ti,ab. OR calcifediol.ti,ab.))

### Web of Science

TS = ((diabet* AND neuropath* AND (“vitamin D” OR cholecalciferol OR ergocalciferol OR calcifediol))

### AND (Random* OR Trial* OR Placebo* OR “Controlled Study”))Cochrane Library (CENTRAL)

((diabet*):ti,ab,kw AND (neuropath*):ti,ab,kw

### AND (“Vitamin D” OR Cholecalciferol OR Ergocalciferol OR Calcifediol):ti,ab,kw) CINAHL (via EBSCOhost)

We combined Cochrane RCT specific search filters with the following terms.

((diabetes mellitus OR diabet*)

AND (peripheral neuropathy OR neuropath*)

AND (“vitamin D” OR cholecalciferol OR ergocalciferol OR calcifediol))

### EBSCO (Cross‐Database Search)

((diabet*) AND (neuropath*)

AND (“vitamin D” OR cholecalciferol OR ergocalciferol OR calcifediol)

AND (random* OR placebo* OR “clinical trial” OR “intervention study”)) Google Scholar

“vitamin D” AND diabetes AND neuropathy AND prevention AND (“randomized controlled trial” OR RCT OR placebo)
